# Protocol for the biofabrication of immunocompetent tumor-on-chip models from patient solid tumors for assessment of anticancer therapies

**DOI:** 10.1016/j.xpro.2025.103895

**Published:** 2025-06-14

**Authors:** Martin Nurmik, Ségolène Ladaigue, Isabella Hofer, Auriane Debache, Irina Veith, Fatima Mechta-Grigoriou, Stephanie Descroix, Gerard Zalcman, Maria Carla Parrini

**Affiliations:** 1 Institut Curie, INSERM U1339, Stress and Cancer Laboratory, PSL Research University, Paris, France; 2 Institut Roche, Boulogne-Billancourt, France; 3 Institut Curie, CNRS UMR168, Laboratoire Physico Chimie Curie, Institut Pierre-Gilles de Gennes, PSL Research University, Paris, France; 4 Université Paris Cité, CIC INSERM 1425, Thoracic Oncology Department, Hospital Bichat-Claude Bernard, Paris, France

**Keywords:** Cancer, Cell culture, Cell isolation, Immunology, Tissue Engineering

## Abstract

We present a protocol to generate immunocompetent 3D tumor-on-chip models from human solid tumors, enabling more accurate therapy response assessment than traditional 2D assays. We outline the isolation and culture of autologous tumor cells, CD8^+^ tumor-infiltrating lymphocytes, and cancer-associated fibroblasts, followed by their encapsulation in a 3D biomimetic matrix within microfluidic devices and subsequent video microscopy. The protocol is adaptable to other tumor types, including breast and colon cancer.

For complete details on the use and execution of this protocol, please refer to Veith et al.^1^

## Highlights

• Establishment of primary tumor, CAF, and T cell cultures from patient samples
• Steps for the encapsulation of autologous cells in primary tumor-on-chips (ToCs)
• Guidelines for using primary immunocompetent ToCs for assessment of therapy efficacy

## Abstract (editor highlights)

Publisher’s note: Undertaking any experimental protocol requires adherence to local institutional guidelines for laboratory safety and ethics.

## Graphical abstract

**Figure:**
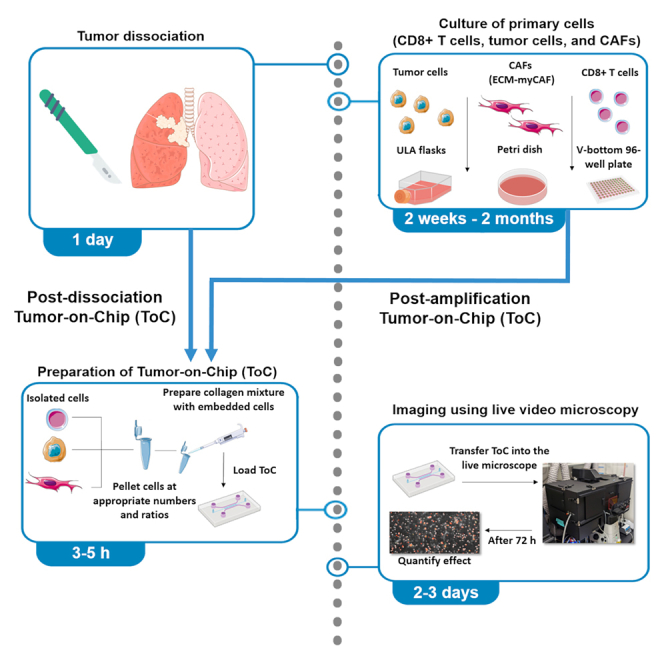

## Before you begin

Here, we introduce a protocol designed for the development of patient-derived autologous tumor-on-chips (ToCs), utilizing a readily accessible commercial microfluidic device (AIM-Biotech, #DAX-1), with the main objective of assessing therapy efficacy. While tailored for primary patient tissue utilization, the described ToC model can be easily adapted to any cell type (e.g., human or murine, primary or immortalized, transfected or genetically edited).

The protocol outlined below enables the creation of a multipopulational, immunocompetent, 3D ToC model using relatively low cell counts (20,000 – 40,000 cells in total), suitable for small surgical samples (range size of 1-2 cm^3^). Additionally, this protocol is well-suited for the quick reconstitution of the patient’s cell co-cultures immediately after surgery, preserving the highest possible degree of similarity to *in vivo* cell identities and properties.

This protocol outlines two tumor-on-chip (ToC) approaches: **post-dissociation** and **post-amplification** ToCs.

**Post-dissociation ToCs** are assembled immediately after tumor resection and typically involve cell types that can be isolated at relatively high yields, such as epithelial tumor cells and CD8^+^ T cells. For this approach, the isolated cells can be directly utilized following primary tumor dissociation. Users can proceed straight to the “Encapsulation of primary cells in collagen-based tumor-on-chips (ToCs)” section of the protocol if freshly isolated cells are used.

**Post-amplification ToCs** are recommended for cell types that are isolated in lower numbers from the tumor sample, such as cancer-associated fibroblasts (CAFs) or cells which are difficult to isolate with a high degree of purity. In these cases, cultivation or amplification of the cells is necessary before their incorporation into the ToC. This protocol provides detailed methods for amplifying or maintaining cultures of CAFs, epithelial tumor cells, and CD8^+^ T cells for short times (from 2 weeks up to 2 months). However, for cell types not addressed in this protocol, alternative culturing methodologies may need to be applied.

### Institutional permissions

Fresh tumor samples were provided by the Pathology department of the University Bichat Hospital (AP-HP) and by the Institut Mutualiste Montsouris (IMM) in Paris, France, from patients with NSCLC having undergone standard-of-care surgical resection. Tissue samples were taken from surgical residues available after histopathological analysis and not required for diagnosis. Patients were informed of the potential use for research of their samples/data and signed an informed non-opposition form according the French regulation on non-interventional studies using leftover biological specimens for research. The human experimental procedures follow the Declaration of Helsinki guidelines. Ethical approval was obtained from the institutional review board of the French Society of Respiratory Medicine (Société de Pneumologie de Langue Française, SPLF) (number CEPRO 2020-051) and from the Institutional Review Board and Ethics Committee of Institut Curie Hospital group (CRI-DATA190154 and CRI-DATA230112). Specimens at IMM were collected under a dedicated protocol approved by the French Ethics and Informatics Commission (EUdract 2017-A03081-52).

## Key resources table

**Table:** 

REAGENT or RESOURCE	SOURCE		IDENTIFIER
**Chemicals, peptide, and recombinant proteins**			

rIL-2	Gibco		#PHC0021
Collagen type I, rat tail	Thermo Fisher Scientific		#A1048301
Human EGF recombinant protein	Thermo Fisher Scientific		#PHG0311
Human FGF-basic (FGF-2/bFGF) recombinant protein	Thermo Fisher Scientific		#13256-029
TrypLE Express Enzyme	Gibco		#12605010
Hydrocortisone	Sigma-Aldrich		#H0888-1G
B-27 Supplement (50X); serum free	Thermo Fisher Scientific		#17504044
Heparin	Sigma-Aldrich		#H3149-10KU
Human AB serum	Institut Jacques Boy, Reims, France		#201021334
Insulin (human)	Sigma-Aldrich		#I9278-5ML
Sodium pyruvate	Thermo Fisher Scientific		#11-360-070
Sodium hydroxide solution	Sigma-Aldrich		#S2770
Penicillin/Streptomycin	Thermo Fisher Scientific		#15140122
PBS (10x)	Gibco		#12579099
PBS (1x)	Gibco		#10010023
Distilled water	Gibco		#15230162
RPMI-1640 medium	Sigma R8758		#SH30027.01
Fetal bovine serum	Biosera		#FB-1001/500

**Critical commercial assays**			

Tumor Dissociation Kit; human	Miltenyi Biotec		#130-095-929
Tumor Cell Isolation Kit; human	Miltenyi Biotec		#130-108-339
CD8 Microbeads	Miltenyi Biotec		#130-045-201
Debris removal solution	Miltenyi Biotec		#130-109-398
Dead Cell Removal Kit	Miltenyi Biotec		#130-090-101
CellTrace Yellow Cell Proliferation Kit	Thermo Fisher Scientific		#C34573
CellTrace Far Red Cell Proliferation Kit	Thermo Fisher Scientific		#C34572
CellTrace CFSE Cell Proliferation Kit	Thermo Fisher Scientific		#C34570
CellEvent Caspase-3/7 Detection Reagent	Thermo Fisher Scientific		#C10423
DRAQ7	Invitrogen		#D15106

**Biological samples**			

Tumor sample (NSCLC)	Bichat-Claude Bernard Hospital (AP-HP)		Veith et.al^1^

**Software and algorithms**			

ImageJ	Schneider et al., 2012, https://doi.org/10.1038/nmeth.2089	https://imagej.nih.gov/ij/	
MetaMorph	Molecular Devices	https://www.moleculardevices.com/products/cellular-imaging-systems/high-content-analysis/metamorph-microscopy	
SpatioTemporal Apoptosis Mapper (STAMP)	Veith et al., 2018	Veith et al.^5^	
TM-STAMP	University of Rome TorVergata	Veith et.al^1^	
MATLAB R2022b	MathWorks	https://www.mathworks.com/products/matlab.html	

**Other**			

DMEM F12 medium	Gibco	#11330-032	
DMEM, high glucose	Sigma	#D6429	
Dimethyl sulfoxide (DMSO)	PanReac AppliChem	# A3672,0100	
Ultra-low attachment 75 cm^2^ U-flask	Corning	#3814	
Ultra-low attachment plates (6-well)	Corning	#3471	
96-well V-bottom plate	Greiner	#651261	
Petri dish; PS; 94/16 mm	Greiner	#353003	
Falcon 100 μm cell strainer	Corning	#352360	
idenTx 3 microfluidic chips	AIM Biotech	#289DAX-1	
QuadroMACS separator	Miltenyi Biotec	#130-090-976	
LS columns	Miltenyi Biotec	#130-042-401	
MS columns	Miltenyi Biotec	#130-042-201	
Synth-a-Freeze cryopreservation medium	Gibco	#A1254201	
pH-indicator strips, pH 6.5–10.0	Merck Millipore	#109543	
Mineral oil	Sigma-Aldrich	#M8410	
CoolCell LX	Corning	#432138	
Mr. Frosty freezing container	Thermo Fisher Scientific	#5100-0001	

## Materials and equipment

**Table:** Medium A

Reagent	Final concentration	Amount
RPMI 1640	88.9%	44.45 mL
Penicillin streptomycin	0.1 %	0.05 mL
10× sodium pyruvate	1%	0.5 mL
Human serum	10%	5 mL
**Total**		**50 mL**

Store at 4°C for up to 1 month.

**Table:** Fibroblast Media

Reagent	Final concentration	Amount
DMEM, high glucose	89%	450 mL
Decomplemented FBS	10%	50 mL
P/S	1%	5 mL
**Total**		**500 mL**

Store at 4°C for up to 1 month.

**Table:** Spheroid Media (Stock)

Reagent	Final concentration	Amount
DMEM-F12	97%	485 mL
P/S	1%	5 mL
B27 Mix	0.1x	1 vial (50X)
Heparin	4 μg/mL	Variable (based on stock concentration)
Human Insulin	5 ng/mL	Variable (based on stock concentration)
Hydrocortisone	1 μg/mL	Variable (based on stock concentration)
**Total**		**500 mL**

Store at 4°C for up to 1 month.

**Table:** Spheroid Media (Final) – Make 50 mL aliquots

Reagent	Final concentration	Amount
Spheroid Media	100%	50 mL
EGF	20 ng/mL	Variable (based on stock concentration)
FGF-basic	20 ng/mL	Variable (based on stock concentration)
**Total**		**50 mL**

Store at 4°C for up to 2 weeks.

## Step-by-step method details

### Isolation and culture of primary tumor cells

**Timing: for tumor cell isolation: 3–4 h**

**Timing: of tumor cell culture: 2 weeks up to 2–3 months**

This section covers the isolation of primary tumor cells and their culture as non-adherent spheroids for post-amplification ToC experiments. Culture conditions are aimed at maximizing cellular proliferation, amplification and maintenance (2–3 months) of the tumor spheroids for replicate experiments and/or freezing.

***Note:*** Any tumor cell isolation method from primary samples is compatible with this culture protocol. An efficient isolation protocol for lung and other solid tumors can be found in Corgnac et al.^2^ and this was the protocol that we applied for the isolation of our samples. In our case, we used the Tumor Cell Isolation Kit (Miltenyi Biotec, #130-108-339) according to the manufacturer’s instructions.

***Note:*** Prepare all the relevant media before this step. Spheroid Media (Stock) can be stored for up to 1 month, while Spheroid Media (Final) should be used within one week after preparation. All media should be pre-warmed at 37°C for 20 min before use.

1. Centrifuge the tumor cell containing fraction in a centrifuge for 10 min (400–500 g).
2. Remove the supernatant completely.
3. Resuspend the resulting cell pellet in 12 mL of Spheroid Media (Final).
4. Transfer the cells into a T75 Ultra-Low-Attachment Flask (Corning, #3814).

***Optional:*** With lower cell counts (less than 5 × 10^5^ cells), Ultra-Low-Attachment (ULA) well-plates (Corning, #3471) may also be used to increase the spheroid formation efficiency in early stages. In this case, simply resuspend the cells in 3 mL of Spheroid Media (Final) and add the cells to one well of a 6-well ULA plate.

**Figure 1:**
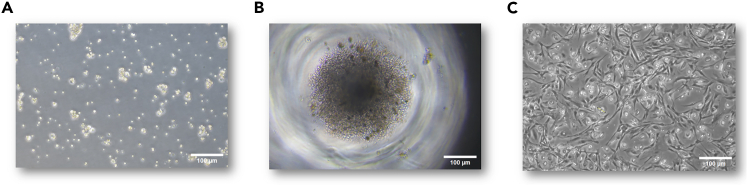
Representative images of primary cultures after one week of culture *(*A) A representative image of a primary spheroid culture (NSCLC). (B) A representative image of a primary CD8^+^ T cell culture. (C) A representative image of a primary cancer-associated fibroblast culture. Depicted cellular density is the approximate confluence of CAFs required before passaging. All cell cultures depicted above were derived from the same tumor.

***Note:*** A representative image of tumor cell culture at Day 7 can be seen in Figure 1.

5. Transfer the flask into a 37°C, 5% CO_2_ incubator for future culture. Cells can then be cultured and fed with spheroid media (final) every two days.

***Optional:*** Lower oxygen concentrations (e.g., 1 to 5% O_2_) can be used to further improve spheroid growth efficiency, but are not strictly necessary.

Spheroids are dissociated for passaging when their diameter reaches approximately 300–400 micrometers or for further experimental use.

6. Transfer the spheroids suspension into a 50 mL tube, and centrifuge at 350 g for 5 min.
7. Remove the supernatant.
8. Add 3–5 mL of PBS (Gibco, #10010023) to the cell pellet to remove any residual media and repeat the centrifugation step (350 g for 5 min).
9. Remove the supernatant and resuspend the cells in 1 mL of TrypLE Express Enzyme (Gibco, #12605010).
10. Vigorously pipette the spheroids up and down 20 times using a P1000 pipette in order to mechanically dissociate the spheroids as much as possible.
11. Transfer the cells to a 37°C incubator for 3 min.
12. Repeat the previous mechanical dissociation step (Step 9) by vigorously pipetting the spheroids up and down 20 times using a P1000 pipette in order to dissociate the spheroids as much as possible.
13. Add 3 mL of Spheroid Media (Final) to the cells to inactivate the TrypLE.
14. Centrifuge the cells at 350 g for 5 min.
15. Remove the supernatant and resuspend the cells in Spheroid Media (Final) or in any other required assay media.

***Note:*** Tumor-derived primary tumor cell cultures can be cultured under both hypoxic (1.5% O_2_) and normoxic conditions (20% O_2_).

***Note:*** Spheroids can also be frozen and defrosted for later use. In this case, the spheroid pellet can be resuspended in 0.5–1 mL of Synth-a-Freeze Cryopreservation Medium (Gibco, #A1254201), placed in a cell storage box (such as Corning CoolCell LX or Mr. Frosty Isopropanol box), kept shortly at −80°C and transferred into liquid nitrogen for long-term storage.

### Isolation and culture of CD8^+^ tumor-infiltrating lymphocytes

**Timing: for CD8**^**+**^**T cell isolation: 3–4 h**

**Timing: of CD8**^**+**^**T cell culture: 1–2 weeks**

This section covers the culture establishment of CD8^+^ tumor-infiltrating lymphocytes and their expansion for follow-up experiments.

***Note:*** Any CD8^+^ T cell isolation method from primary samples is compatible with this culture protocol. Again, we used the protocol by Corgnac et al.^2^ which uses the CD8^+^ Microbeads (Miltenyi Biotec, #130-045-201), applied according to manufacturer’s instructions.

***Note:*** Prepare all relevant media (Medium A) before this step. All media should be pre-warmed at 37°C for 20 min before use.

16. Count the number of CD8^+^ cells and their viability using an automatic cell counter or a hemocytometer.
17. Centrifuge the CD8^+^ cell fraction for 10 min at 400–500 g.
18. Remove the supernatant.
19. Resuspend the cells in requisite amount of Medium A, supplemented with 30 ng/mL of rIL-2 (Gibco, #PHC0021), to obtain approximately 5000 CD8^+^ TILs cells per 100 μL of media.
20. Seed 200 μL of CD8^+^ cell suspension (5000 cells/100 μL) per well into a V-bottomed 96-well plate (Greiner, #651261) and place the plate into a standard 37°C, 5% CO_2_ incubator.

***Note:*** IL-2 containing cell culture media should be replaced weekly. As CD8^+^ TILs are suspension cells, there is no need for trypsinization. For cell expansion, cells are collected, centrifuged (10 min at 400–500 g) and replated at 5000 cells/100 μL density.

***Note:*** Tumor-derived primary CD8^+^ cell cultures should be cultured under 20% O_2_, as lower oxygen densities can hamper T cell proliferation.

***Note:*** For long-term storage, TILs can be frozen in medium A containing IL-2, supplemented with 10% DMSO. Cells are then transferred into −80°C freezers using cryoboxes, followed by later transfer into liquid nitrogen for long-term storage.

***Note:*** A representative image of CD8^+^ T cell culture at Day 7 can be seen in Figure 1B.

### Isolation and culture of cancer-associated fibroblasts

**Timing: for CAF isolation: 3–4 h**

**Timing: of CAF culture: 2–3 weeks**

This section covers the isolation and cultivation of primary cancer associated fibroblasts (CAFs) for culture establishment and follow-up experiments. Based on the expertise of our laboratory on investigating CAF heterogeneity, we note that the here isolated and cultured CAFs correspond to the CAF-S1 subpopulation, specifically the FAP+ ECM-myCAF subset.^3^^,^^4^

***Note:*** Any CAF isolation method from primary samples is compatible with this culture protocol.

***Note:*** Prepare all relevant media before this step. Fibroblast media can be stored for up to one month. All media should be pre-warmed at 37°C for 20 min before use.

21. Centrifuge the non-tumor cell fraction in a centrifuge for 10 min (400–500 g).
22. Remove the supernatant.
23. Resuspend the cells in the appropriate amount of fibroblast media and plate 200,000-500,000 cells per petri dish (Greiner, #353003)
24. Transfer the dish into a 37°C, 5% CO_2_ incubator for future culture. Change fibroblast media every two days.

***Note:*** Lower oxygen concentrations (e.g., 1 to 5% O_2_) can be used to further improve fibroblast growth efficiency, but are not strictly necessary.

***Note:*** When primary CAFs reach 80% confluence (an example can be seen in Figure 1C), they are passaged as follows:

25. Remove the supernatant from the petri dish.
26. Wash the cells using 10 mL of PBS.
27. Remove the PBS washing buffer.
28. Add 1 mL TrypLE Express Enzyme onto the cells.
29. Transfer the cells into an 37°C incubator for 5 min.
30. Verify the detachment of CAFs under a microscope.

***Note:*** If cells do not detach, the side of the petri dish can be tapped by hand to provide additional mechanical stress to detach the fibroblasts.

31. Add 9 mL of fibroblast media to the cells to inactivate the TrypLE enzyme.

***Note:*** If the cells have still not detached, the neutralizing media suspension can be used to vigorously wash the adherent cells in order to provide additional mechanical stress to force cellular detachment.

32. Transfer the cell suspension into a 15 mL tube and centrifuge it for 3–5 min at 350 g.
33. Remove the supernatant and resuspend the cells in the desired volume.
34. Count the number of CAFs and their viability using an automatic cell counter or a hemocytometer for further expansion or storage.
35. For continuous culture, we recommend seeding 250,000 cells per 10-cm dish.

***Note:*** CAFs can be frozen in fibroblast medium, supplemented with 10% DMSO. Cells are then transferred into −80°C freezers using cryoboxes, followed by later transfer into liquid nitrogen for long-term storage.

### Encapsulation of primary cells in collagen-based ToCs

**Timing: 80–110 min**

This section covers the encapsulation of primary cells in collagen gels within commercial microfluidic devices (AIM-Biotech, #DAX-1). The loading volume is 10 μL, comprising the central gel chamber (3.4 μL) and its lateral inlets. After encapsulation, cells can be imaged using any suitable video microscope. The obtained video data can then be used to assess viability, cell death, and other desired parameters.

***Note:*** Humidified boxes filled with distilled water need to be prepared before the start of this step, and transferred to 37°C, 5% CO_2_ incubators for later use.

***Note:*** When necessary, distilled water in the humidified boxes can be supplemented with antibacterial/antimycotic agents to reduce the risk of contamination.

36. Count the number and viability of all desired primary cell populations to be incorporated in the ToC model using an automatic cell counter or a hemocytometer.
37. Calculate the number of required tumor cells for the experiment.

***Note:*** A good compromise to achieve a satisfactory density and convenient image analysis is around 20000–40000 tumor cells per chip (2000–4000 cells per μL of hydrogel, the loading volume being a 10 μL gel). At lower density, the sparse population will limit data acquisition, while at higher density, cellular aggregation can complicate analysis.

***Note:*** It is recommended to prepare an excess of cell-matrix mixture (around 1.2–1.5X) to compensate for pipetting errors or improperly formed hydrogels. E.g. If seeding for six conditions at 30000 cells per each 10 μL gel, then 270000 cells should be set aside (30000 x 6 x 1.5) which should be resuspended in 90 μL of gel mixture (10 x 6 x 1.5).

38. Calculate the number of required CD8^+^ TILs and/or CAF cells.

***Note:*** The cell-to-cell ratio can vary depending on the experiment. Median proportions *in vivo* are 1:3 for TIL/cancer cell ratio and 1:6 for CAF/cancer cell ratio, as assessed by immunostainings of NSCLC samples (n=12).^1^

39. Transfer the required quantity of cells into 1.5 mL tubes and centrifuge for 10 min (400–500 g).

***Note:*** Use one tube per condition, e.g. monocultures or co-cultures, without or with treatment.

40. Completely aspirate the supernatant from all tubes.

***Note:*** At this point, cells can be stained with dyes for the purpose of fluorescent tracking of specific cellular populations (e.g. fluorescent staining of tumor/CAF/CD8^+^ cells) according to their respective manufacturer protocols. Notable dyes that can be used at this step are the CellTrace family of dyes from Invitrogen (#C34573, #C34572, and #C34570).

***Note:*** If cellular staining is carried out at this step, it is recommended to recount the cells after staining to ensure correct co-culture seeding ratios.

**CRITICAL:** Pipette tips, pipettes, and the DAX-1 chips themselves should be transferred into the cold room (4°C–8°C) 30 min before this step to be pre-cooled when used. This is to avoid premature polymerization of the collagen hydrogel due to temperature differences and to ensure reproducibility of polymerized gel texture.

**CRITICAL:** Be extremely careful not to aspirate the cell pellet at this stage.

41. Once cells have been pelleted down and the supernatant has been removed, transfer the cell pellet tubes on ice.
42. Prepare all reagents for collagen gel formation in aliquots in 1.5 mL tubes on ice. These reagents are: 10x PBS (Gibco, #12579099), 1 M NaOH solution (Sigma-Aldrich, #S2770), distilled H_2_O (Gibco, #15230162), and Type I rat tail collagen (3 mg/mL, Thermo-Fisher, #A1048301).

***Note:*** Once prepared, all reagents should be kept on ice.

***Note:*** An additional 1.5 mL tube for the collagen mixture itself (for each condition) should also be prepared and kept on ice.

43. Transfer the ice box with all the cells and reagents into a cold room (4°C–8°C) for all subsequent gel formation steps.
44. Formulate a 2.0–2.5% collagen hydrogel mixture according to the manufacturer’s instructions. An example calculation for a 2.5% Collagen, Rat Tail I (Thermo-Fisher Scientific, #1048301) hydrogel can be seen below:
  a. Volume of collagen needed (V1) + Volume of 10X PBS needed (V2) + Volume of 1N NaOH needed (V3) + Volume of dH_2_O needed (V4).
    i. V1 = [Final conc. of collagen × Total Volume (Vt)] / Initial conc. of collagen.
    ii. V2 = Total Volume (Vt)/10.
    iii. V3 = V1∗0.025.
    iv. V4 = Total Volume (Vt) – (V1 + V2 + V3).
  b. For a 1 mL, 2.5% collagen hydrogel the relevant component concentrations are:
    i. V1 = 833 μL.
    ii. V2 = 100 μL.
    iii. V3 = 20.825 μL.
    iv. V4 = 46.175 μL.

***Note:*** It is recommended to formulate at least 220 μL, preferably 1 mL, of collagen solution to buffer for pH variability that may occur in smaller volumes.

**CRITICAL:** Collagen stock properties can greatly vary between manufacturers and batches. Before experimental implementation, it is recommended to prepare a pre-test collagen mixture, and verify the accuracy of its pH (optimally around 7) using pH indicator strips (Merck-Millipore, #109543). In the case of inaccurate pH, adjust NaOH volume in 0.5 μL increments until correct pH is achieved and keep this adjustment for all experiments with this batch of collagen.

45. Resuspend cell pellets in the required amount of collagen (10 μL per gel), while considering the amplification factor applied earlier. In the case of the cell population that was highlighted earlier (30000 cells per 10 μL) the volume of collagen used would be: 10 μL x 6 (Number of gels) x 1.5 (Amplification factor) = 90 μL

***Note:*** Make sure to dissociate your pellets fully by pipetting the collagen mixture up and down 20-30 times. Avoid too vigorous pipetting, as this may introduce bubbles into the gel mixture, hampering loading and subsequent imaging.

**Figure 2:**
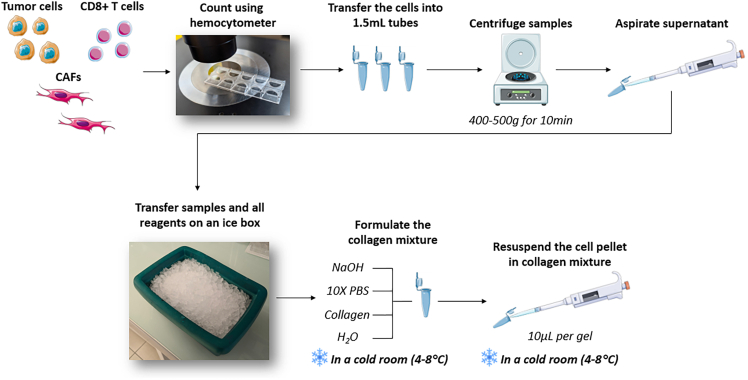
Summary of steps 36–45

***Note:*** A graphical summary of steps 36–45 can be seen in Figure 2.

46. Load the gel chambers on the chip using both inlets. Each gel chamber can hold 10 μL of hydrogel. First, load 2/3 of the gel from the inlet on one side (visually assessed), then carefully load the remaining 1/3 from the other side.

**CRITICAL:** Do not load the gel too forcefully or rapidly, as this can induce the leakage of the gel into the lateral medium channels making this chip unusable.

***Note:*** Samples should be kept on ice while loading the chamber to reduce the speed of polymerization as much as possible.

47. Under the microscope, verify that all loaded gels are intact (no visible bubbles, no leakage of hydrogel into the lateral media channels).

***Note:*** For best cellular viability and ToC reproducibility, it is recommended to load the samples relatively quickly after preparing the cell-collagen mixture. Do not load more than 6–9 gels at any given time. If more chips are needed, it is better to work sequentially.

48. Transfer the ToCs into a 37°C, 5% CO_2_ incubator for 25 min inside the prepared humidified box to allow for hydrogel polymerization.

***Note:*** The humidified chamber is necessary to avoid any potential drying out of the hydrogel, as only small volumes of collagen are used.

49. Remove the ToCs from the incubator.

***Note:*** At this point, specific dyes, e.g., apoptosis reporters such as CellEvent Caspase-3/7 (Invitrogen, #C10423), or total cell death reporters such as DRAQ7 (Invitrogen, #D15106), can be added to the media that is to be added onto the cells.

50. Add 130 μL of media into the two lateral medium channels flanking each gel.

***Note:*** The specific media used can vary according to which cell types are incorporated into the ToCs. When isolating and culturing lung tumor cells, CAFs, and CD8^+^ T cells, we recommend using IL-2 supplemented Medium A, since this media is best tolerated by all of the three cell types.

51. After adding media, add a drop of sterile mineral oil (Sigma-Aldrich, #M8410) on top of the inlets of gel and media channels to minimize evaporation.
52. At this point, ToCs are ready for subsequent imaging.

***Note:*** A video summary can be seen in Methods video**(**summarization of steps 46–52).

Methods Video S1. Video summary; related to steps 46–52

## Expected outcomes

**Figure 3:**
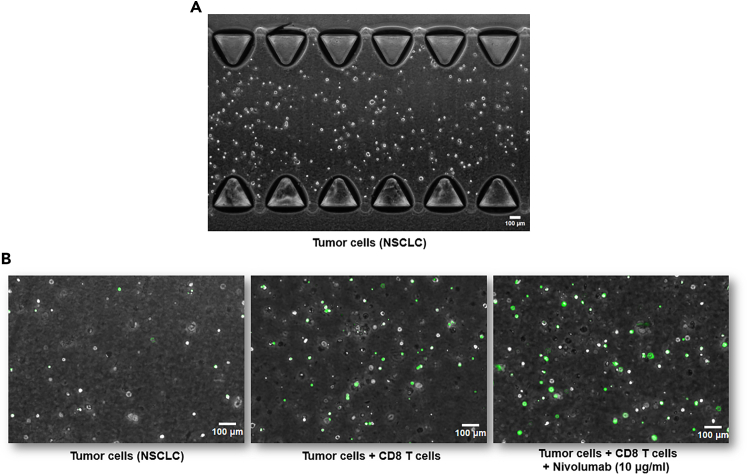
Expected outcomes in regards to therapy response and gel loading (A) An representative image of a correctly loaded ToC with primary NSCLC cells under video microscopy (5X objective). (B) An example of primary immunotherapy response in autologous ToC at the 30 h time point. Cells have been treated with CellEvent Caspase-3/7 Detection Reagent, with fluorescent signal (green) indicating apoptosis activation.

Following this protocol, we were able to create fully autologous primary, immunocompetent tumor-on-chips, composed of autologous CAFs, TILs, and tumor cells, which allowed us to assess patient-specific immunotherapy responses using video-microscopy. As drugs or target compounds can be simply added to media channels, it enables for assessment of a variety of different therapeutics and their effects on co-cultured cell populations.^1^ A representative figure illustrating immunotherapy response in primary NSCLC cells, as well as an example image of a correctly loaded ToC model can be seen in Figure 3. For data analysis of imaging data, we recommend using automated image analysis tools such as STAMP and TM-STAMP, as described in our previous publications.^1^^,^^5^

In addition to live imaging, two types of follow up experiments can be carried out on primary ToCs post-imaging. Firstly, hydrogels can be digested and cells can be extracted from ToCs for flow cytometry analysis or single-cell sequencing (scRNA-seq) at assay endpoint. To obtain sufficient amounts of cells for subsequent experiments (e.g. scRNAseq), several identical gels can be combined per condition to reach the sufficient number of cells required.

Secondly, hydrogels can be extracted from the chip by removing the bottom membrane that typically allows for gas exchange. Gels can then be embedded in formalin-fixed, paraffin-embedded (FFPE) blocks, allowing for immunohistochemistry (IHC) staining for target proteins.

## Limitations

As the number of gels that can be imaged under a single video-microscope is relatively limited (12 maximum), the current protocol cannot be conveniently applied to high-throughput compound screening. Furthermore, depending on the tumor type, obtaining a high number of viable primary cells (especially epithelial tumor cells) can be challenging, limiting the number of ToCs than can be generated and the number of treatments that can be tested for each patient. In our experience, cellular densities of tumors can vary quite considerably; for example, it is far easier to generate long-term primary cultures from non-small cell lung cancers than breast tumors, as the cell yield efficiency from lung tumor samples is several times higher than from breast tumor samples.

## Troubleshooting

### Problem 1

When placing the ToC under the video-microscope for live microscope (Step 52), cell viability rapidly decreases, leading to many unspecific dead events that are not related to the tested compounds.

### Potential solution

In the idenTx 3 chips, gas exchange (CO_2_, O_2_) occurs through the bottom membrane of the chip. We recommend not to directly mount ToCs on the microscope stage holder or on glass slides, but to slightly elevate the chip using magnets, as described in Veith, et al., 2021 (Supplementary Figure 5).^5^

Similarly, while most live imaging microscopes are equipped with humidity control, micro-evaporation can still pose a significant challenge, particularly given the small volumes of media typically used in the reservoirs (130 μL). To mitigate media evaporation, we recommend adding distilled H_2_O to the empty spaces, between the gel inlets within the chip. Additionally, placing sterilized and water-saturated sponges inside the microscope chamber can help maintain a humid environment and further reduce the evaporation rate.

### Problem 2

Tumor cell cultures are not viable in long term culture, but rather can only be maintained for a limited time (few weeks) and do not expand (Step 15).

### Potential solution

This is likely due to the small amount of epithelial tumor cells post isolation. The density of tumor tissue can vary significantly based on the tissue of origin, ranging from extremely dense such as lung tumors (approx. 30–40 x 10^6^ cells per 1 × 1 cm piece post-dissociation) to low density tumors such as breast tumors (approx. 1–3 x 10^6^ cells per 1 × 1 cm piece post-dissociation). We therefore recommend using the largest surgical samples available, even if a necrotic core is present.

To enhance the viability of isolated cells from tumor samples with high levels of necrosis, we recommend using the Debris Removal Solution (Miltenyi Biotec #130-109-398) followed by the Dead Cell Removal Kit (Miltenyi Biotec #130-090-101), in that order, as per manufacturer’s instructions. These steps should be performed after the initial tumor dissociation but prior to isolating specific cell populations. Implementing this procedure is expected to significantly improve the viability of the isolated cells.

### Problem 3

Fluorescent signals are lost over long-duration (72 h) imaging (Step 52).

### Potential solution

A large number of cell dyes become cumulatively weaker as cells divide. This can lead to the loss of the fluorescent signal. We recommend testing different cell dyes at different concentrations, in order to identify the most suitable one for each specific cell population. However, one should consider that higher dye concentrations may also affect cell viability, as many dyes are dissolved in cytotoxic agents such as DMSO.

### Problem 4

Cells form clusters, microspheres, or aggregates inside the ToC (especially when generated from spheroid cultures) impairing automated image analysis and quantification (Step 52).

### Potential solution

This is indicative that the dissociation process was not complete and that the seeded cell population was not a single cell suspension. To avoid this, the cell suspension can be filtered through a 50 μm strainer before seeding. If this is not sufficient, a strainer with a smaller pore size can also be used to exclude cellular aggregates.

## Resource availability

### Lead contact

Further information and requests for resources and reagents should be directed to and will be fulfilled by the lead contact, Martin Nurmik (martin.nurmik@curie.fr).

### Technical contact

Technical questions related to the execution of this protocol should be directed to and will be fulfilled by the technical contact, Martin Nurmik (martin.nurmik@curie.fr).

### Materials availability

This protocol does not include unique materials.

### Data and code availability

This protocol does not include datasets.

## Acknowledgments

The project was supported by Fondation ARC pour la Recherche sur le Cancer (PGA1 RF20180206991, PGA12021010002992_3578, 4th year PhD fellowship to I.H.); INSERM (ITMO 3R no. 19CR046-00, ITMO Equipment 2016, ITMO Equipment no. 22CQ036-01, ITMO MIC DYNAMO no. 23CM020-00; ITMO PCSI HT on Chip no. 23CP060-00; Programme Impulsion MecaCell3D); Fondation Chercher et Trouver, Agence Nationale de la Recherche under the France 2030 program (PEPR MED-OOC, TME-On-Chip, no. ANR-24-EXME-0005); a postdoctoral fellowship of Fondation de France to M.N. (no. 00149014/WB-2023-50799); and a CIFRE fellowship to I.V., funded in part by the Association Nationale de la Recherche et de la Technologie (ANRT) on behalf of the French Ministry of Higher Education and Research and in part by Institut Roche. This work had financial support from ITMO Cancer of Aviesan within the framework of the 2021–2030 Cancer Control Strategy with funds administered by INSE. This project received funding also from the European Union’s Horizon 2020 program under the Marie Skłodowska-Curie grant agreement no. 847718 and from Horizon Europe program under the Arturo project grant agreement no. 101136464, funded by the European Union. Views and opinions expressed are, however, those of the author(s) only and do not necessarily reflect those of the European Union or the Health and Digital Executive Agency. Neither the European Union nor the granting authority can be held responsible for them. Graphical abstract and figures were created with the help of Biorender.com.

## Author contributions

Conceptualization, M.N. and M.C.P.; methodology, M.N., I.V., S.D., M.C.P., and A.D.; investigation, M.N., S.L., I.H., and I.V.; supervision, F.M.-G., S.D., G.Z., and M.C.P.; writing – original draft, M.N.; writing – review and editing, M.N., S.L., I.H., A.D., G.Z., and M.C.P.

## Declaration of interests

I.V. was a Roche employee. F.M.-G. received research support from Roche, Institut Roche, and AstraZeneca.

## References

[1] Veith I., Nurmik M., Mencattini A., Damei I., Lansche C., Brosseau S., Gropplero G., Corgnac S., Filippi J., Poté N. (2024). Assessing personalized responses to anti-PD-1 treatment using patient-derived lung tumor-on-chip. Cell Rep. Med..

[2] Corgnac S., Lecluse Y., Mami-chouaib F. (2021). Isolation of tumor-resident CD8+ T cells from human lung tumors. STAR Protoc..

[3] Kieffer Y., Hocine H.R., Gentric G., Pelon F., Bernard C., Bourachot B., Lameiras S., Albergante L., Bonneau C., Guyard A. (2020). Single-cell analysis reveals fibroblast clusters linked to immunotherapy resistance in cancer. Cancer Discov..

[4] Costa A., Kieffer Y., Scholer-Dahirel A., Pelon F., Bourachot B., Cardon M., Sirven P., Magagna I., Fuhrmann L., Bernard C. (2018). Fibroblast Heterogeneity and Immunosuppressive Environment in Human Breast Cancer. Cancer Cell.

[5] Veith I., Mencattini A., Picant V., Serra M., Leclerc M., Comes M.C., Mami-Chouaib F., Camonis J., Descroix S., Shirvani H. (2021). Apoptosis mapping in space and time of 3D tumor ecosystems reveals transmissibility of cytotoxic cancer death. PLoS Comput. Biol..

